# Supplementary material for: The effect of maternal HMB supplementation on bone mechanical and geometrical properties, as well as histomorphometry and immunolocalization of VEGF, TIMP2, MMP13, BMP2 in the bone and cartilage tissue of the humerus of their newborn piglets
Source: PLoS One. 2021 Feb 24;16(2):e0240642. doi: 10.1371/journal.pone.0240642 (PMC7904207; doi:10.1371/journal.pone.0240642)
Supplement: Attachment — Submitted filename: Response to reviewers.docx [file pone.0240642.s001.docx]

**Reviewer 1**

Thank you very much for your review. All comments contributed to the introduction of valuable changes to the manuscript. The work was supplemented with two additional analyzes that are used to evaluate the expression of proteins in tissues in immunohistochemical methods. The first of these methods was the method consisting in assessing the participation of cells with positive responses to the presence of VEGF, TIMP2, MMP13, BMP2. This analysis consisted in designating 10 fields of observation in which all cells were counted. Then, cells with a positive reaction to the presence of tested proteins were counted and their percentage in relation to all cells was calculated. The second method used to study the expression of VEGF, TIMP2, MMP13, BMP2 in the matrix of growth plate cartilage and bone trabeculae was the analysis of gray, which is based on the brightness of pixels. The higher the pixel value, the weaker the expression of the tested protein. Both methods confirmed the previously performed observation that HMB supplementation influences to the expression of VEGF, TIMP2, MMP13, BMP2 both in the growth plate cartilage and the humerus bone trabeculae. Both methods used to analyze the expression of the studied proteins, both in cells and in the matrix of bone and cartilage tissue, are standard methods used in studies of the expression of these proteins. Both methods are standard methods used in studies of the expression of proteins.

In the presented manuscript, the results of the research obtained in our study were compared with the results published by Blicharski et al. in 2017. In this publication the effects of maternal supplementation on the prenatal development of the femur of their offspring were tested on the same model. The comparison of the results shows that the humerus responds differently to the changes in maternal nutrition, which may be due to the fact that the prenatal development of this two long bones is other. The obtained results are new as there is no literature information regarding the influence of maternal HMB supplements on the prenatal development of the humerus and they complement the available knowledge.

**Reviewer 2**

Thank you for your review. All comments have been incorporated in the revised manuscript.

Detailed comments:

1. The method of euthanizing animals has been completed

2. All tables were complete for standard deviation

3. The numbering of figures and gaps in the text were checked

4. The discussion was shortened by 1,5 pages
